# Predictive value of MRI features on glioblastoma

**DOI:** 10.1007/s00330-023-09535-x

**Published:** 2023-04-05

**Authors:** Xiaodong Ma, Jiayu Liu

**Affiliations:** Department of Neurosurgery, General Hospital of Chinese PLA, 28th Fuxing Road, 100853 Beijing, China

Glioblastoma multiforme (GBM) is the most common primary intracranial malignancy, and its treatment is a global challenge [1]. Its low survival rate and high recurrence rate bring great harm to patients. It is urgent to find a better treatment plan for GBM. Early and accurate diagnosis is significant for formulating the treatment plan and prognosis prediction of GBM. Non-invasive methods to monitor treatment efficacy for GBM after surgery will help patients receive appropriate treatment and thus contribute to their long-term survival. Imaging examinations are an intuitive and objective way to diagnose GBM and an essential way to predict its prognosis. In particular, MRI provides a factual basis for the clinical development of GBM treatment, not only for the preoperative analysis of tumour morphology but also for monitoring the effect and sensitivity of glioma treatment, such as radiotherapy, chemotherapy and targeted therapy [2].

It has been widely accepted and applicable to observe the size and morphology of intracranial tumours and distinguish the malignant degree of glioma by imaging technology. In recent years, many researchers have believed that imaging indicators have a specific value in predicting the prognosis of GBM patients. The tumour’s location, size, degree of necrosis and oedema, rate of tumour progression and degree of tumour enhancement are closely related to the prognosis. MRI has a high spatial resolution and is irreplaceable in observing tumour features. In MRI sequences, 3D T1-weighted sequences can directly evaluate tumour characteristics. Diffusion tensor imaging (DTI) was used for tracer reconstruction of white matter fibre bundles. Functional magnetic resonance imaging (fMRI) was used for tracer reconstruction of the visual cortex, language area and other critical functional areas. Magnetic resonance venography (MRV) mode was used to reconstruct important arteries in the surgical area. Magnetic resonance venography (MRV) mode was used to reconstruct essential veins in the surgical area. And magnetic resonance spectroscopy (MRS) was used to reconstruct the tumour metabolic area [3]. These MRI sequences have been widely used to establish the initial diagnosis, operative plan and post-treatment monitoring. And many studies have shown a specific correlation between the survival rate of patients and some indicators provided by MRI [4]. Lacroix et al [5] believed that tumour necrosis, enhancement and oedema in preoperative MRI examination were related to the prognosis of GBM patients. At the same time, the patient’s gender, tumour site, preoperative tumour volume, mass effect and treatment method did not significantly correlate with the patient’s prognosis. As measured by MRI, the extent of resection (EOR) positively influences overall survival in patients with glioma, which is an important independent prognostic factor. In addition, diffusion-weighted imaging (DWI) can provide partial information in prognostic prediction. DWI can determine whether the diffusion movement of water molecules in the tumour is limited. Studies have shown that the elevation of postoperative DWI signal in the tumour area can be used as one of the independent prognostic factors. Moreover, the recurrence sites of GBM were outside the hyperintensity zone on postoperative DWI. Apparent diffusion coefficient (ADC) and relative cerebral blood volume (rCBV) can also provide a prognostic prediction for patients undergoing chemoradiotherapy at an early stage. Susceptibility-weighted imaging (SWI) is considered helpful for monitoring the efficacy of GBM after comprehensive treatment. SWI can predict a patient’s sensitivity to combination therapies, such as surgery and chemoradiotherapy, and thus provide clinicians with information for personalised treatment. In the preoperative SWI sequence, the hypointensity of the lesion is correlated with the prognosis. A large SWI hyperintensity lesion is considered more sensitive to antiangiogenic therapy, cytotoxic therapy and radiation therapy, indicating a good prognosis. MRS-related studies have shown that changes in NAA and Cho values can be used as imaging biomarkers to assess response to antiangiogenic drugs. MRS may also help to distinguish pseudoprogression from tumour recurrence. In perfusion-weighted imaging (PWI), rCVB can reflect the tumour’s blood supply, judge the degree of glioma malignancy and play a specific role in differentiating tumour recurrence from radiation necrosis [6]. However, for predicting the prognosis of GBM, it is necessary to use a personalised imaging analysis protocol and comprehensive analysis of the results of multiple sequences. GBM has different manifestations on MRI, indicating that the images shown in MRI contain information at the cell or gene level, which significantly impacts the evaluation of tumour treatment effects and prognosis prediction.

Bevacizumab, tumour angiogenesis for treating GBM, is mainly used with other therapies (chemotherapy, radiation therapy and immunotherapy) for GBM patients [7]. Bevacizumab is not a recommended treatment in the NCCN guidelines, Central Nervous System Cancers (Version 3.2020). It was only effective against tumour oedema and radiation necrosis, and although it can keep progression-free survival, it cannot prolong overall survival in GBM patients [8]. In addition, bevacizumab inhibited neovascularization at an early stage, but the vascular mimicry (VM) increased 6 days after treatment [9]. The increase in VM after bevacizumab administration may be responsible for treatment resistance. A study recently published in *European Radiology* showed that angiogenic habitats (perfusion- and vessel size–derived vascular habitats) could predict time to progression (TTP) in recurrent glioblastomas treated with antiangiogenic therapy [10]. The results showed that MRI features have profound clinical implications for aiding patient stratification for antiangiogenic therapy in recurrent GBM. However, the results of this innovative study, published in *European Radiology*, can be used to monitor response to bevacizumab but cannot be used to assess prognosis. In addition, since bevacizumab can only inhibit neovascularization at an early stage, the MRI features of angiogenic habitats in this study also need further studies to prove.

In further studies, more factors affecting GBM need to be included and combined with comprehensive MRI sequences, and more valuable research results can be obtained. Furthermore, it is difficult to determine whether bevacizumab or other therapies achieve the treatment effect in this study. It is also necessary to analyse this issue. In addition, the high proliferation of malignant tumour cells increases the transfer of amino acids into cells and the synthesis of proteins. PET/CT or PET/MRI with 11C-methionine has become a vital imaging examination method for the diagnosis and differential diagnosis of glioma. Therefore, if PET/CT or PET/MRI can be combined, more parameters may be found to evaluate the effect of bevacizumab treatment or other tumour angiogenesis treatments in GBM patients. In summary, MRI features play an essential role in the prognosis assessment and treatment outcome evaluation of GBM. Further multicentre prospective studies are still necessary to further demonstrate its clinical value.

## Declarations

### Guarantor

The scientific guarantor of this publication is Xiaodong Ma.

### Statistics and biometry

No complex statistical methods were necessary for this paper.

### Methodology

• Editorial comment
